# The late-follicular-phase progesterone to retrieved oocytes ratio in normal ovarian responders treated with an antagonist protocol can be used as an index for selecting an embryo transfer strategy and predicting the success rate: a retrospective large-scale study

**DOI:** 10.3389/fendo.2024.1338683

**Published:** 2024-05-15

**Authors:** Hongxia Zhang, Shuo Yang, Lixue Chen, Caihong Ma, Ping Liu, Jie Qiao, Rong Li

**Affiliations:** Center for Reproductive Medicine, Department of Obstetrics and Gynecology, Peking University Third Hospital, Beijing, China

**Keywords:** *in vitro* fertilization, late follicular phase, progesterone level, pregnancy outcome, antagonist protocol

## Abstract

**Objective:**

To determine whether the late-follicular-phase progesterone to retrieved oocytes (P/O) ratio during *in vitro* fertilization (IVF)/intracytoplasmic sperm injection (ICSI) impacts pregnancy outcomes.

**Design:**

12,874 cycles were retrospectively categorized into four groups according to the P/O ratio percentile, with divisions at the 25th, 50th and 75th percentiles.

**Results:**

The clinical pregnancy and live birth rates of fresh cycle embryos in Group D were significantly lower than those in the other three groups (45.1% and 39.0%, 43.2% and 37.2%, 39.6% and 33.5%, 33.4% and 28.2% in Group A, B, C, D, respectively; both P < 0.008). Multivariate logistic regression analysis revealed a significant negative correlation between the P/O ratio and live birth, particularly when the P/O ratio was ≥0.22 (OR = 0.862, 95% CI [0.774–0.959], P = 0.006).

**Conclusions:**

The P/O ratio has certain predictive value for IVF/ICSI pregnancy outcomes and can be used for decision-making decision regarding fresh embryo transfer.

## Introduction

According to the World Health Organization (WHO), infertility and sterility will be the third most serious condition worldwide in the 21st century. Furthermore, it is estimated that 15% to 20% (40–50 million) of women of reproductive age in China suffer from infertility, and 822,246 assisted reproductive technology (ART) cycles were conducted between 1981 and 2011 (1). Although IVF/ICSI is an effective method for treating infertility, overcoming the pregnancy rate bottleneck has always been a research hotspot in the field of reproductive medicine, and a premature surge in serum progesterone (P) in the late follicular phase may significantly influence the success rate of IVF/ICSI. Late follicular phase progesterone elevation (LFPE) is defined as a premature increase in serum P on the day of human chorionic gonadotropin (hCG) injection, and in controlled ovarian stimulation (COS) cycles, the incidence of LFPE ranges from 4.5 to 30% (2, 3). Whether LFPE affects the pregnancy outcomes of fresh IVF/ICSI cycles is still controversial. Some studies have shown that LFPE can reduce the pregnancy rate after embryo transfer in fresh cycles by affecting the quality of oocytes/embryos and endometrial receptivity (4–6). Conversely, another study revealed no correlation between LFPE length and pregnancy outcomes (7, 8).

Currently, the LFPE threshold is usually set according to the effect of LFPE on IVF/ICSI clinical pregnancy outcomes, and the threshold reported in the literature is 2.54–7.95 nmol/L (9, 10), which may be the main reason for the different results of previous studies. Furthermore, retrospective studies often fail to account for various potential confounders, including patient age, ovarian responsiveness, COS protocol, the quantity of oocytes and high-quality embryos, the day of embryo transfer, and the number of embryos transferred. These factors may have contributed significantly to the varying findings reported in previous studies.

The mechanism of LFPE is still unclear. One proposed theory is that in IVF cycles in which the pituitary is not downregulated, the concurrent elevation of luteinizing hormone (LH) levels and the abundance of LH receptor-expressing granulosa cells, attributed to multiple developing follicles, leads to intensified LH signaling and subsequently augmented P production (11, 12). However, LFPE has also been observed in pituitary-desensitized COS cycles (13). Another hypothesis concerns the gonadotropin (Gn) used in COS, and studies have shown that LFPE occurs when COS is induced using both human menopausal gonadotrophin (hMG) and purified follicle-stimulating hormone (FSH) preparations (containing less than 1% LH) (14, 15), even with varying FSH dosages (16). The authors of the latter studies postulated that the number of follicles is intricately linked to LFPE, suggesting that it is a reflection of the number of recruited follicles and is not necessarily indicative of a pathological condition. A retrospective study of 687 infertile women undergoing fresh IVF/ICSI treatment with a long agonist protocol revealed that the P/O ratio may be a valuable tool for predicting IVF outcomes when compared with serum P levels alone (17). The number of studies assessing the relationship between the P/O ratio and pregnancy outcomes among pregnant patients treated with the antagonist protocol is scarce. Thus, in this study, we aimed to explore the predictive value of the P/O ratio for pregnancy outcomes in IVF/ICSI cycles among normal ovarian responders treated with the antagonist protocol.

## Materials and methods

### Patients and study design

This was a retrospective cohort study. The data of patients who underwent fresh IVF/ICSI embryo transfer at Peking University Third Hospital between June 2016 and June 2021 were collected. The inclusion criteria were as follows: ① maternal age between 20 and 40 years without polycystic ovary syndrome (PCOS); ② normal ovarian responders who were undergoing their first IVF/ICSI cycles; ③ patients treated with the gonadotropin-releasing hormone (GnRH) antagonist protocol; and ④ patients who underwent a transfer of 2 fresh day-3 cleavage-stage embryos. The exclusion criteria were ① patients who had undergone preimplantation genetic testing or sperm or egg donation cycles; ② patients with conditions or diseases that may influence the pregnancy rate, including hydrosalpinx, intrauterine adhesion, submucous myoma, endometrial hyperplasia, uterine malformation, or endometrial polyps or an endometrial thickness ≤7 mm; ③ patients with uncontrolled endocrine diseases, such as hyperprolactinemia or thyroid dysfunction; or ④patients with incomplete data.

Based on the available data, the P/O ratio was calculated, and patients were categorized into four groups, with the 25th, 50th and 75th percentile set as the group boundaries and P/O ratios of 0.15, 0.22 and 0.32 set as the cutoff points. The four groups were defined as follows: P/O ratio <0.15 (Group A); 0.15 ≤ P/O ratio < 0.22 (Group B); 0.22 ≤ P/O ratio <0.32 (Group C); and 0.32 ≤ P/O ratio (Group D). According to the results of our previous prospective randomized controlled clinical studies (18, 19), it is recommended that the whole embryo be frozen when P levels are ≥6 nmol/L on the day of hCG injection. Therefore, all patients included in this study had P levels <6 nmol/L on the day of hCG injection.

### Treatment process

Gn [FSH (Merck Serono Company of Switzerland or Merck of America)] and hMG (Livzon Pharmaceutical Company of Zhuhai) (150–225 U/day) were used to induce ovarian stimulation on the 2^nd^ day of menstruation or withdrawal bleeding. The initial dose of Gn was determined according to the following information: antral follicle count (AFC); serum levels of FSH, LH, estradiol (E_2_), P and anti-Müllerian hormone (AMH); age; and body mass index (BMI). After 4–5 days, follicular development was monitored by ultrasonography, and the dosage of Gn was adjusted accordingly. When the diameter of the follicle was larger than 14 mm, 0.25 mg of the antagonist (Merck Serono, Germany) was applied until the trigger day. When two follicles reached a diameter of ≥17 mm, endometrial thickness was measured on the same day, and venous blood was taken to measure LH, E_2_ and P levels. Recombinant hCG (Merck Serono Company of Switzerland) 250 µg or hCG 10000 IU (Livzon Pharmaceutical Company of Zhuhai) was injected that night. After 36–38 hours, the eggs were obtained by puncture under the guidance of vaginal B-ultrasound, and IVF or ICSI was selected according to the semen analysis results. Per the routine of our center, the 2 embryos with the best scores at the cleavage stage (D3) were transferred. Luteal support was started on the day of egg retrieval, and 90 mg of P gel (Merck Serono Company, Switzerland) was given qd, 20 mg of dydrogesterone (Abbott Healthcare Products Company, Netherlands) was given orally bid, or 20 mg of P was given by intramuscular injection qd (Zhejiang Xianxian Pharmaceutical Company).

According to morphology and level of fragmentation, day-3 embryos were divided into four grades (20): Grade 1: 4–6 cells on day 2 or 6–8 cells on day 3, with evenly sized blastomeres without cellular fragments and a smooth cytoplasm without vacuoles; Grade 2: 4–6 cells on day 2 or 6–8 cells on day 3, with <20% fragmentation, unevenly sized blastomeres and/or slightly granulated cytoplasm; Grade 3: >20% but <50% fragmentation, with blastomeres/cells of all sizes and/or heavily granulated cytoplasm or vacuoles; or Grade 4: >50% fragmentation. Embryos with D2 ≥2 cells and grade III or above and embryos with D3 blastomeres ≥5 cells and grade III or above were regarded as available embryos. The P/E_2_ ratio was calculated with the following formula: P (in ng/mL) × 1,000/E_2_ (in pg/mL) (21).

The serum β-hCG level was measured fourteen days after embryo transfer. A positive β-hCG level greater than 25 U/L indicated the likelihood of pregnancy. At 28 to 35 days after embryo transfer, gynecological ultrasonography was conducted. Clinical pregnancy was defined as a gestational sac or primitive cardiac pulsation on ultrasound. The patients were followed up by telephone until pregnancy termination. Miscarriage was defined as a clinical pregnancy that ended before 28 gestational weeks of gestation; early miscarriage was defined as a pregnancy that ended at ≤12 gestational weeks; and late miscarriage was defined as a pregnancy that ended between 13 and 28 gestational weeks. Live birth was defined as delivery of a newborn with vital signs at 28 weeks or more of gestation.

### Observation indicators

The main observation index of IVF/ICSI outcomes was the live birth rate of transplantation cycles (the number of live births/number of transplantation cycles × 100%), and the secondary observation indices were the clinical pregnancy rate (the number of clinical pregnancies/the number of transplantation cycles × 100%), early abortion rate (the number of early abortions/the number of clinical pregnancies × 100%), and implantation rate (the number of intrauterine embryos/the total number of transplanted embryos × 100%).

### Statistical analysis

The SPSS 26 software package was used for statistical analysis. The clinical data of the patients were collected by qualified personnel. Normally distributed data are expressed as the mean ± standard deviation $\left(\bar{\chi }\;\pm \;s\right)$ . Data that conformed to a nonnormal distribution are presented as the median (25th percentile, 75th percentile) [M (P25, P75)], and categorical data are presented as the rate (%). One-way analysis of variance or the Kruskal−Wallis nonparametric test with multiple comparisons and the χ^2^ test were used to analyze continuous data and categorical data, respectively. To adjust for the influence of potential confounders on the live birth rate, binary logistic regression analysis with the likelihood ratio and backward regression was used to analyze related factors.

A two-sided P <0.05 was considered to indicate statistical significance among groups, and a two-sided P <0.008 was considered to indicate statistical significance between two groups of categorical data.

## Results

### Basic patient information

From June 2016 to June 2021, 18,813 women who experienced infertility underwent their first cycles of IVF/ICSI treatment with the antagonist protocol in our center. According to the inclusion and exclusion criteria, 12,874 cycles were included, and 5,939 cycles were excluded. The clinical characteristics of each group are shown in **Table 1**. Age was higher in Group D than in the other three groups (P<0.05). The AMH level in Group D was lower than that in the other three groups, but the baseline FSH level was higher than that in the other three groups (P<0.05). The AFC in Group D was lower than that in each of the other groups (P<0.05). The BMI of patients in Group D was lower than those in Group A and Group D (P<0.05). There were significant differences in the percentage of patients with primary infertility and the percentage of patients with infertility factors (**Table 1**).

**Table 1:** Baseline characteristics of the patients in each group.

Items	Group A	Group B	Group C	Group D	χ2/F	P value
No. of patients	3038	3228	3436	3172		
Age (years)	31.1 ± 3.8	31.5 ± 3.9^a^	32.1 ± 3.9^ab^	32.9 ± 3.9^abc^	131.404	0.000
AMH (ng/ml)	2.8 (2.2–4.2)	2.8 (2.0–3.9) ^a^	2.7 (1.9–3.5) ^ab^	2.7 (1.8–3.2) ^abc^	262.622	0.000
Baseline FSH (IU/L)	6.7 (5.5–7.3)	6.8 (5.5–7.5) ^a^	6.9 (5.6–7.7) ^ab^	6.9 (5.7–8.0) ^abc^	78.380	0.000
Number of antral follicles	12 (10–15)	10 (9–14)^a^	10 (8–12)^ab^	9 (6–11)^abc^	981.794	0.000
BMI (kg/m^2^)	22.5 (20.4–24.8)	22.2 (20.3–24.5) ^a^	21.9 (20.0–24.1) ^ab^	21.7 (19.8–23.9) ^ab^	84.070	0.000
Primary infertility (%)	56.1	58.7	58.5	61.6	19.611	0.000
Infertility factors					79.174	0.000
Female factor	34.2	37.9	40.2	42.8		
Male factor	37.9	35.6	34.8	30.5		
Bilateral factors	22.1	21.4	20.8	22.9		
Unexplained factors	5.8	5.1	4.2	3.7		

a indicates P<0.05 compared with Group A; b indicates P<0.05 compared with Group B; c indicates P<0.05 compared with Group C.

### Ovarian stimulation

The dosage of Gn, duration of Gn, LH, P and P/E_2_ ratio on the day of hCG injection, and the P/O ratio were highest in Group D and lowest in Group A, with significant differences (P<0.05). The E_2_ level on the day of hCG injection and endometrial thickness on the day of hCG injection in Group D were significantly lower than those in the other three groups (P<0.05). Among patients who underwent ICSI, the proportion of MII oocytes was significantly higher in groups D and C than in groups A and B (P<0.05). The ICSI fertilization rate in Group D was significantly lower than those in the other groups (P<0.008). The number of available embryos in Group D was significantly lower than that in the other three groups (P<0.05). The ratio of available embryos to retrieved oocytes was significantly higher in group D than in the other groups (P<0.008) (**Table 2**).

**Table 2:** Ovarian stimulation in the patients in each group.

Items	Group A	Group B	Group C	Group D	χ2/F	P value
No. of patients	3038	3228	3436	3172		
dosage of Gn (U)	1875 (1425–2400)	2100 (1650–2700) ^a^	2325 (1800–2850) ^ab^	2625 (2100–3150) ^abc^	1198.753	0.000
duration of Gn (d)	10.0 ± 1.6	10.1 ± 1.6	10.2 ± 1.5^a^	10.3 ± 1.7^ab^	13.600	0.000
LH on hCG injection day (IU/L)	1.4 (0.9–2.2)	1.8 (1.1–2.8) ^a^	1.9 (1.1–3.1) ^ab^	2.1 (1.2–3.6) ^abc^	454.564	0.000
E_2_ on hCG injection day (pmol/L)	6834 (5110–9382)	6953 (5167–9963)^a^	7164 (5270–10235) ^ab^	6498 (4574–9276) ^abc^	90.621	0.000
P on hCG injection day (nom/L)	1.4 (1.1–1.7)	2.1 (1.6–2.5) ^a^	2.6 (2.0–3.3) ^ab^	3.2 (2.4–4.9) ^abc^	5280.862	0.000
P/E_2_ ratio	0.23 (0.17–0.31)	0.33 (0.24–0.44) ^a^	0.40 (0.31–0.53) ^ab^	0.55 (0.42–0.74) ^abc^	4695.487	0.000
P/O ratio	0.11 (0.09–0.13)	0.18 (0.16–0.20) ^a^	0.26 (0.24–0.29) ^ab^	0.43 (0.37–0.54) ^abc^	12072.402	0.000
Endometrial thickness on hCG day(cm)	10.8 ± 1.4	10.7 ± 1.4	10.6 ± 1.4^ab^	10.6 ± 1.5^ab^	18.199	0.000
No. of oocytes retrieved	13 (11–16)	12 (9–14) ^a^	10 (8–13) ^ab^	7 (5–9) ^abc^	3928.658	0.000
MII oocyte rate (%)	76.3	76.6	79.4 ^ab^	81.0^ab^	118.840	0.000*
ICSI rate (%)	43.9	41.3	41.5	38.4 ^abc^	19.611	0.000*
Available embryo number	4 (3–7)	3 (2–5) ^a^	3 (2–5) ^ab^	2 (2–3) ^abc^	988.474	0.000
Ratio of available embryos to retrieved oocytes (%)	35.9	35.8	36.7	40.9^abc^	106.512	0.000

a indicates P<0.05 compared with Group A; b indicates P<0.05 compared with Group B; c indicates P<0.05 compared with Group C. * indicates a significant difference between the two groups (P<0.008).

### Pregnancy outcomes

The results of the IVF/ICSI procedures are presented in **Table 3**. The implantation rate of fresh embryos, clinical pregnancy rate and live birth rate decreased with increasing P/O ratio, with the lowest rates in Group D (all P<0.008). Both the implantation rate and fresh cycle live yield in Group C were lower than those in Groups A and B, respectively (P < 0.008). The clinical pregnancy rate in Group C was lower than that in Group C (P < 0.008), while there were no significant differences among the remaining groups (P>0.008). There was no significant difference in the early spontaneous abortion rate among the groups (P>0.05).

**Table 3:** Pregnancy outcomes of the patients in each group.

Items	Group A % (n)	Group B % (n)	Group C % (n)	Group D % (n)	χ2	P value	Linear-by-linear association χ2	P value
Implantation rate	31.4 (1907/6067)	29.9 (1928/6456)	26.7 (1834/6872)^ab^	22.5 (1427/6344)^abc^	110.069	0.000*	95.731	0.000
Clinical pregnancy rate	45.1 (1371/3038)	43.2 (1395/3228)	39.6 (1362/3436)^b^	33.4 (1060/3172)^abc^	103.923	0.000*	97.615	0.000
Early spontaneous abortion rate	10.2 (140/1371)	10.2 (142/1395)	12.1 (165/1362)	12.7 (135/1060)	6.457	0.091	5.558	0.018
Fresh cycle live yield	39.0 (1185/3038)	37.2 (1202/3228)	33.5 (1151/3436)^ab^	28.2 (893/3172)^abc^	87.870	0.000*	72.448	0.000

a indicates P<0.05 compared with Group A; b indicates P<0.05 compared with Group B; c indicates P<0.05 compared with Group C. * indicates a significant difference between the two groups (P<0.008).

**Figure 1:**
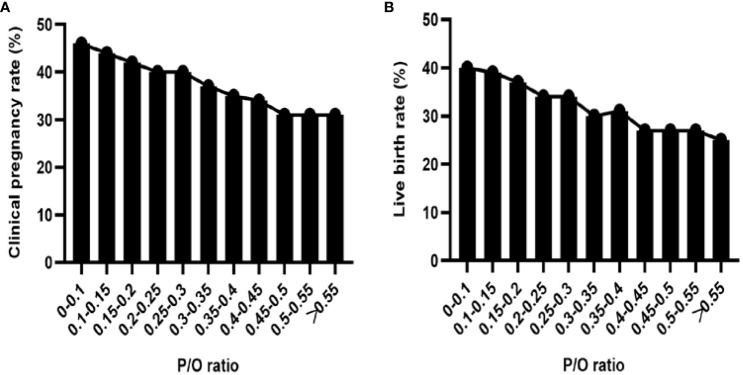
**(A)** Clinical pregnancy rate of subgroups with different P/O ratios. **(B)** Live birth rate in each subgroup according to the P/O ratio.

A subgroup analysis was conducted on the P/O ratio at a significance level of 0.05. The results revealed a negative correlation between P levels and both the clinical pregnancy rate and live birth rate (**Figure 1**). Linear-by-linear association analysis demonstrated a significant decrease in the implantation rate, clinical pregnancy rate, and fresh-cycle live birth rate with an increase in the P/O ratio (*P*=0.000). Conversely, there was a significant increase in the early spontaneous abortion rate with increasing P/O ratio (P=0.018, **Table 3**).

### Multivariate binary logistic analysis of factors affecting live births

Univariate binary logistic regression analysis showed that age, BMI and P/O ratio were negatively correlated with live births (P<0.05), but AFC, the AMH level, the LH level and endometrial thickness on hCG injection day, the number of oocytes retrieved and available embryo number were positively correlated with live births (P<0.05), the basic FSH level, infertility factors, primary infertility, E_2_ on hCG injection day, the dosage of Gn, duration of Gn and ICSI were not significantly correlated with live births (P>0.05) (**Supplementary Table 1**).

Multivariate binary logistic regression analysis with the likelihood ratio and backward regression was conducted to assess potential influencing factors, including age, baseline FSH, AMH, AFC, BMI, primary infertility status, infertility factors, duration of Gn, Gn dosage, LH and E_2_ levels on hCG injection day, endometrial thickness, the number of available embryos, and the P/O ratio. Age, BMI and the P/O ratio were negatively correlated with the number of live births (P<0.05) but that the AFC, available embryo number, LH level and endometrial thickness on the hCG injection day were positively correlated with the number of live births (P<0.05) (**Table 4**).

**Table 4:** Multivariate binary logistic regression analysis of factors affecting live birth.

Influencing factor	P value	OR (95% CI)
Age (years)	0.000	0.957 (0.948–0.967)
AFC	0.009	1.012 (1.003–1.021)
BMI (kg/m^2^)	0.000	0.978 (0.968–0.989)
P/O ratio	0.000	
P/O ratio <0.15		Reference
0.15 nmol/L ≤ P/O ratio <0.22	0.514	0.966 (0.869–1.073)
0.22 ≤ P/O ratio <0.32	0.006	0.862 (0.774–0.959)
P/O ratio ≥ 0.32	0.000	0.717 (0.637–0.806)
LH on hCG injection day (IU/L)	0.000	1.087 (1.067–1.107)
Endometrial thickness on hCG day (cm)	0.000	1.127 (1.098–1.157)
Available embryo number	0.000	1.064 (1.048–1.080)

## Discussion

In this study, the linear-by-linear association results showed that the implantation rate, clinical pregnancy rate and fresh-cycle live birth rate decreased with increasing P/O ratio. Furthermore, multivariate binary logistic regression analysis confirmed these findings, indicating a significant negative correlation between the P/O ratio and live births, particularly when the P/O ratio was ≥0.22.

LFPE is influenced by numerous other factors. Recently, it has also been reported that the degree of P elevation varies with the type of ovarian response. Among the poor responders, the LFPE levels were consistently lower, averaging 1.5 ng/ml. Intermediate responders had slightly higher levels, averaging 1.75 ng/mL, while high responders had the highest LFPE levels, averaging 2.25 ng/mL (22). In previous study, the ovulation induction protocol was shown to be is one of the factors. Compared to the antagonist protocol, the GnRH agonist protocol was found to be associated with a substantial increase in P levels (≥ 6.2 nmol/L) (23). To ensure the reliability of our findings, we limited our study to infertile patients with a normal ovarian response who underwent the antagonist protocol.

LFPE was found to be positively correlated with the E_2_ level on the day of hCG injection but not with the pregnancy rate (24). Further study on the relationship between LFPE and E_2_ revealed that there was no significant threshold value for the trigger-day P/E_2_ ratio that was beneficial for predicting a live birth of GnRH antagonist cycles (25); however, in another study, P/E_2_ > 0.55 affected the clinical pregnancy rate of women undergoing long agonist protocols and cleavage-stage but not blastocyst-stage embryo transfer (26). Given the positive correlation between E_2_ and P levels, which may negatively impact endometrial receptivity, the predictive value of the P/follicle ratio for ART outcomes was evaluated in another study. In a group of 8649 normal responders, a LFPE-to-follicle ratio ≥14 mm was superior to the LFPE-to-follicle ratio alone in the prediction of clinical pregnancy (27). Due to potential variations in the interpretation of ultrasound examination results among observers, some authors have utilized the number of oocytes retrieved as a replacement for the number of follicles. In this study, the authors assessed the ability of the P/O ratio to predict ART outcomes in 687 infertile women undergoing treatment with long agonist protocols, fresh day-3 or day-5 embryos were transferred. The results indicated that the detrimental cut-off value for the P/O ratio was >0.15, with a sensitivity of 62% and specificity of 61%. Patients with a P/O ratio ≤0.15 had a significantly higher pregnancy rate (35.3%, [p < 0.001]) than did patients with a P/O ratio >0.15 (18.8%). A prospective study including 200 patients who underwent surgery with a long agonist protocol and whose embryos were transferred on day 3 or day 5 revealed that the P/MII oocyte ratio was significantly lower in patients who achieved clinical pregnancy than in those who could not, and using a cutoff value of 0.125, the sensitivity and specificity of the P/MII ratio in the prediction of no pregnancy in IVF/ICSI were 75.7% and 77.1%, respectively, with the area under the receiver operating curve (ROC-AUC) = 0.808 (28). We also found similar results in patients treated with the antagonist protocol in the present study. However, the results from a retrospective study including 6157 patients with agonist or antagonist COH revealed that in a multivariate analysis, the P/O ratio was not significantly associated with live birth but that P was independently associated, suggesting that the P/O ratio added no additional predictive value to the two variables separately and that the number of follicles or oocytes did not protect against the negative impact of P on live birth rates (29). This difference may be due to the older age of the patients (median age of 35 years), the percentage of embryos transferred on day 3 or day 5, and the number of embryos transferred. A previous prospective randomized controlled study in our center showed that the implantation rate, clinical pregnancy rate and live birth rate of fresh cycle embryo transfer in patients with an hCG injection day P ≥6 nmol/L were significantly reduced but that the pregnancy outcomes of fresh-cycle blastocyst transfers were significantly better than those of D3 embryo transfers (18). Therefore, in the current study, we focused solely on D3 embryo transfer data for our analysis.

FSH actively promotes P synthesis and output from granulosa cells without luteinization by upregulating the expression and increasing the enzymatic activity of 3β-hydroxysteroid dehydrogenase (3β-HSD), which converts pregnenolone to P (30). A correlation may exist between the number of hormonally active follicles and LFPE; thus, patients with more follicles usually have LFPE (9). However, from this study, we found the opposite result: in the group with the highest P/O ratio, the P level on the day of hCG injection was the highest, but the number of oocytes retrieved was the lowest, and vice versa. Interestingly, the numbers of retrieved oocytes and available embryos were lower in the group with the highest P/O ratio than in the other three groups; however, the percentages of MII oocytes were similar, and the ratio of available embryos to retrieved oocytes was the highest among the groups. These findings suggest that the group with the highest P/O ratio may have greater potential to develop into available embryos, which is consistent with the results of these studies (31–33). These authors reported that LFPE had no impact on oocyte/embryo quality. However, the implantation rate, clinical pregnancy rate and live birth rate of fresh-cycle pregnancies were significantly lower in the group with an increase in the P/O ratio than in the other groups, which supports the detrimental effect of LFPE on pregnancy outcomes via its effect on the endometrium (34). It was found that LFPE may change the endometrium from the proliferative phase to the secretory phase in advance, resulting in the unsynchronized development of the endometrium and embryo and subsequently affecting embryo implantation (35, 36). This was reinforced by evidence showing changes in endometrial gene expression. There were 140 gene disorders in the endometrium on the day of hCG injection in the P>4.77 nmol/L group compared with the P<4.77 nmol/L group (37), and LFPE can inhibit HOXA10 by promoting the expression of miR-135a, thus changing the expression of related genes and affecting endometrial receptivity (38).

Previous studies have focused on the detrimental effects of LFPE on clinical pregnancy and live birth, but in our study, the relationship between the P/O ratio and early spontaneous abortion was explored. Although the early spontaneous abortion rate tended to increase with increasing P/O ratio, the difference was not significant, which corroborates previous observations (39).

The main strengths of this study include the large sample size and adjustment for potential confounders, such as patient age, ovarian response, COS protocol, day of embryo transfer, and number of embryos transferred. Moreover, PCOS is a prevalent endocrine disorder characterized by a diverse range of clinical phenotypes, including hyperandrogenemia, menstrual disorders and polycystic ovary morphology. Patients with PCOS exhibit increased sensitivity to Gn, leading to higher follicle production than in normal individuals (40), that is associated with adverse pregnancy outcomes, such as a low embryo implantation rate, clinical pregnancy rate, and live birth rate of fresh cycle embryo transfer, as well as an elevated miscarriage rate (41). Consequently, individuals afflicted with PCOS were excluded from this study. The main limitation arises from its retrospective nature. Despite the use of strict inclusion criteria regarding patient age and ovarian function, significant differences in basic characteristics persist among the groups. In the group with the highest P/O ratio, patients were older, had higher basic FSH levels, and had fewer antral follicles. These factors may account for the longer COS duration, higher Gn dosage, and lower number of retrieved oocytes. The age range of patients was too large, that may have an impact on pregnancy outcome. Additionally, this study did not differentiate the effects of hMG and purified FSH on pregnancy outcomes, which may impact the likelihood of LFPE (14, 15).

In conclusion, the rates of implantation, clinical pregnancy, and live birth in fresh-cycle embryo transfer decreased progressively with an increase in the P/O ratio, reaching significance when the ratio was ≥0.22. Based on these findings, we postulate that the P/O ratio has predictive value for pregnancy outcomes in IVF/ICSI procedures. Therefore, whether to carry out fresh embryo transfer in patients with LFPE and few retrieved oocytes should be carefully considered. However, due to the retrospective design of this study, randomized trials on potential biological mechanisms are necessary to further investigate the impact of the P/O ratio on embryo development and endometrial receptivity in the future.

## Data availability statement

The original contributions presented in the study are included in the article/**Supplementary Material**. Further inquiries can be directed to the corresponding authors.

## Ethics statement

The studies involving humans were approved by the Reproductive Medicine Ethics Committee of Peking University Third Hospital. Ethics No.: 2018SZ-001. The studies were conducted in accordance with the local legislation and institutional requirements. The participants provided their written informed consent to participate in this study.

## Author contributions

HZ: Data curation, Formal Analysis, Investigation, Methodology, Writing – original draft. SY: Formal Analysis, Methodology, Writing – review & editing. LC: Data curation, Writing – review & editing. CM: Writing – review & editing. PL: Writing – review & editing. JQ: Writing – review & editing. RL: Formal Analysis, Funding acquisition, Methodology, Writing – review & editing.
