# Supplementary material for: The late-follicular-phase progesterone to retrieved oocytes ratio in normal ovarian responders treated with an antagonist protocol can be used as an index for selecting an embryo transfer strategy and predicting the success rate: a retrospective large-scale study
Source: Front Endocrinol (Lausanne). 2024 May 15;15:1338683. doi: 10.3389/fendo.2024.1338683 (PMC11133602; doi:10.3389/fendo.2024.1338683)
Supplement: Supplementary file 1 [file Table_1.doc]

Supplementary table I Univariate binary logistic regression analysis of factors affecting live births

| Influencing factor | P value | OR（95% CI） |
| --- | --- | --- |
| Age (years) | 0.000 | 0.948（0.939-0.956） |
| AMH(ng/ml） | 0.000 | 1.043（1.024-1.061） |
| Basic FSH (IU/L) | 0.560 | 1.005（0.988-1.022） |
| AFC | 0.000 | 1.035（1.027-1.044） |
| BMI（kg/m^2^） | 0.002 | 0.983（0.973-0.994） |
| P/O ratio | 0.000 |  |
| P/O ratio <0.15 |  | Reference |
| 0.15 nmol/L ≤P/O ratio <0.22 | 0.150 | 0.928(0.838-1.027) |
| 0.22 ≤P/O ratio <0.32 | 0.000 | 0.788(0.712-0.870) |
| P/O ratio≥ 0.32 | 0.000 | 0.613(0.551-0.681) |
| Infertility factors | 0.099 |  |
| Unexplained |  | Reference |
| Female factor | 0.266 | 0.905（0.758-1.079） |
| Male factor | 0.984 | 0.998（0.836-1.192） |
| Mixed factors | 0.909 | 0.989（0.824-1.189） |
| Primary infertility | 0.454 | 0.922（0.874-1.014） |
| LH on hCG injection day (IU/L) | 0.000 | 1.060（1.041-1.078） |
| E_2_ on hCG injection day (pmol/L) | 0.844 | 1.002（0.980-1.025） |
| Endometrial thickness on hCG injection day(cm) | 0.000 | 1.146（1.041-1.078） |
| No. of oocytes retrieved | 0.000 | 1.013（1.005-1.022） |
| ICSI fertilization | 0.111 | 0.942（0.874-1.014） |
| dosage of Gn (U) | 0.844 | 1.002（0.980-1.025） |
| duration of Gn (d) | 0.073 | 0.831（0.678-1.018） |
| available embryo number | 0.000 | 1.085（1.070-1.101） |
